# BSr_3_ Superalkali as a Promising Catalyst for Ambient Nitrogen Reduction: A Pathway toward Efficient Ammonia Synthesis

**DOI:** 10.1021/prechem.5c00284

**Published:** 2026-03-12

**Authors:** Natalia Wiszowska, Natalia Rogoża, Celina Sikorska

**Affiliations:** † Faculty of Chemistry, University of Gdańsk, Fahrenheit Union of Universities in Gdańsk Wita Stwosza 63, 80-308 Gdańsk, Poland; ‡ Department of Physics, 1415 The University of Auckland 38 Princes Street, Auckland 1010, New Zealand

**Keywords:** molecular clusters, ab initio calculations, catalyst, electroreduction, nitrogen reduction reaction (NRR)

## Abstract

The transformation of the earth-abundant dinitrogen molecule (N_2_) into ammonia (NH_3_) under mild conditions remains a fundamental challenge in chemistry. The BSr_3_ superatom designed in our previous work (Wiszowska N, Rogoża N, Sikorska C, *Phys. Chem. Chem. Phys.* (2025), **27**: 23468–23486) has been used as a catalyst for the conversion of molecular nitrogen into ammonia. Owing to its low ionization energy (4.033 eV), the BSr_3_ transfers electron density to adsorbed N_2_, partially populating antibonding π* orbitals, lowering the triple N≡N bond order and initiating N–N bond elongation. Subsequent proton-coupled electron-transfer steps further elongate the N–N distance in a stepwise manner, establishing a correlation between structural distortion and the progressive weakening of the N≡N bond, ultimately facilitating bond cleavage. The catalytic conversion of N_2_ to NH_3_ catalyzed by BSr_3_ proceeds via an associative distal pathway, with initial hydrogenation at the nitrogen atom coordinated to strontium atoms (N^Sr^), followed by release of the first NH_3_ molecule upon N–N bond rupture and subsequent hydrogenation of the second nitrogen (N^B^, bound to a boron atom of the BSr_3_ cluster). The release of the NH_3_ molecule is the potential-determining step, with a corresponding free energy uphill of 0.28 eV. The preceding exergonic hydrogenation steps help offset the energy cost of NH_3_ desorption, ensuring overall thermodynamic favorability. These findings position the BSr_3_ superalkali cluster as a promising noble-metal-free catalyst for sustainable ammonia synthesis under ambient conditions.

## Graphical abstract

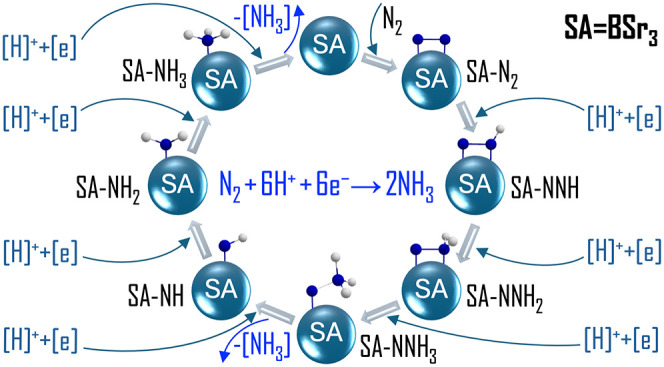

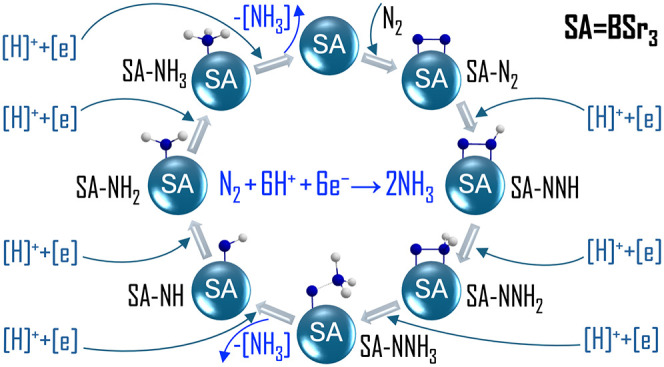

## 1 Introduction

Superatoms are an emerging, rapidly expanding field in materials science that brings together the physics of electronic structure with the chemistry of atomically precise molecular clusters.^1–3^ Materials composed of superatoms, the so-called cluster-assembled solids, offer the promise of atomic precision, high tunability, and a robust material framework.^3–8^ Superalkalis are a class of superatoms characterized by low ionization energies (IEs) and might serve as reducing agents in chemical processes. In 1982, Gutsev and Boldyrev proposed the simple formula ML_k+1_ to describe mononuclear superalkalis, in which M denotes a k-valent electronegative central atom coordinated by k+1 alkali-metal atoms (L).^9^ Examples of mononuclear superalkalis include FLi_2_,^10,11^ OLi_3_,^12,13^ and CLi_5_.^14^ The existence of OL_3_ (L = Li, Na, K), ML_2_ (M = F, Cl, Br, I; L = Li, Na, K), SLi_3_, Li_3_F_2_, Li_2_CN, and Na_2_CN superalkalis has been confirmed by experiments.^15^ Since the early 1980s, a lot of effort has been devoted to introducing alternative superalkali species, including polynuclear N_4_Mg_6_M (M = Li, Na, K) superalkalis,^16^ star-like CBe_5_Au_5_^+^ superalkali cation,^17^ organic heterocyclic molecules,^18^ superalkali molecules containing halogenoids,^19,20^ organophosphine-ligated transition-metal cluster Ni_9_Te_6_(P­(C_2_H_5_)_3_)_8_,^21,22^ and superalkalis with a boron atom acting as a central atom.^23–25^ The superalkali systems, with high thermodynamic stability and low ionization energies, can act as electron donors in reducing counterparts with low electron affinities (EAs).^3,16,25,26^

Superalkalis can be explored as catalysts for dinitrogen activation (EA of −1.8 eV) and conversion to ammonia (NH_3_). The Haber-Bosch process is the primary source of the world’s ammonia production (approximately 175 million metric tons) and accounts for more than 90% of the annual production. Despite significant efforts in optimizing the Haber-Bosch process, it still consumes from 1 to 2% of the energy consumed by the chemical industry worldwide because the production process requires high temperatures (at 573–873 K) and pressures (at 100–360 atm), currently producing more than 1.6% of global carbon dioxide emissions.^27^ Hence, developing efficient catalysts that convert nitrogen molecules into ammonia is crucial for mitigating the growing energy crisis and combating global warming. However, the high stability of N_2_ makes conversion difficult. This is a challenging transformation as the dinitrogen molecule has a N≡N triple bond and a high activation energy (941 kJ/mol).^28^ Considering the above challenges, in this study, a superalkali system as a potential candidate for a catalyst for dinitrogen conversion to ammonia is investigated.

Although several [superalkali]­[dinitrogen] complexes have been reported,^16,26,29,30^ there is a need to investigate their subsequent chemical transformations under mild conditions, especially if these processes are to be performed catalytically. In our recent work, we systematically investigated a family of boron–alkaline earth superalkali clusters (BAe_3_, Ae = Be, Mg, Ca, Sr, Ba) using an integrated first-principles approach combined with quantitative structure–property relationship (QSPR) modeling.^25^ This study demonstrated that BAe_3_ systems possess exceptionally low ionization energies, with BBa_3_ exhibiting the lowest IE (3.82 eV) among the investigated clusters, making them strong electron donors. These clusters were shown to activate inert molecules, such as CO_2_ and N_2_, by forming either BAe_3_–**Y** charge–transfer complexes or covalent BAe_3_ **Y** (Y = CO_2_, N_2_) species.^25^ The stability and geometry of these [BAe_3_]­[**Y**] species were found to correlate strongly with the ionization energy and dipole moment of the superalkali, establishing a predictive design principle for reducing agents. Specifically, by strategically tuning the BAe_3_ cluster composition, it is possible to engineer low-abundance and low-cost alkaline-earth-metal-based electron donors for sustainable chemical applications (including nitrogen fixation). Against this backdrop, the BSr_3_ cluster emerges as a particularly compelling candidate, combining a favorable electronic structure with strong reducing power to enable efficient N_2_ activation under ambient conditions, which is a critical step toward sustainable ammonia synthesis.^25^

The BAe_3_ (Ae = Be, Mg, Ca, Sr, Ba) superalkali clusters can be highly efficient in weakening the triple bond in molecular nitrogen.^25^ The mixed BAe_3_ clusters, such as BBe_2_Mg, readily react with N_2_ to form BAe_3_N_2_ molecules, where the nitrogen atoms become strongly bound.^25^ However, the substantial reducing power of these clusters and the large binding energy of the resulting complexes often render the nitrogen atoms inaccessible for further transformation.^25^ In contrast, the nonmixed BAe_3_ clusters, particularly BSr_3_, facilitate N_2_ activation by forming BAe_3_–N_2_ complexes featuring B–N and B–Ae bridging modes. Within these BAe_3_ clusters, although BBa_3_ exhibits the lowest ionization energy, the catalytic efficiency for N_2_ reduction cannot be inferred solely from ionization energy. Excessive spatial diffuseness in larger clusters (due to the large ionic radius of Ba atoms) may weaken the effective orbital overlap with the π* antibonding orbital of N_2_, thereby decreasing directional π-backdonation. In turn, BSr_3_ provides an optimal balance between strong electron-donating capability and geometric compactness. With a low ionization energy (4.03 eV) and a more confined electronic density distribution, the BSr_3_ promotes efficient charge transfer while preserving favorable orbital alignment with the N_2_ antibonding orbital.^25^

Computational studies demonstrate that the BSr_3_ efficiently transfers electronic charge to adsorbed small molecules, enabling the activation of chemically inert N_2_ through significant electron donation.^25^ Its low ionization energy and structural compactness collectively position BSr_3_ as a uniquely balanced member of the BAe_3_ family. The BSr_3_ sufficiently reduces N_2_, yet it is structurally defined enough to avoid excessive stabilization of intermediates. This combination of reducing strength and mechanistic controllability provides a compelling rationale for exploring BSr_3_ as a molecular catalytic center for ammonia synthesis. Unlike the energy-intensive Haber–Bosch process and the costly use of noble-metal catalysts, BSr_3_ offers a pathway to ammonia synthesis under ambient conditions, representing a paradigm shift toward sustainable, economically viable nitrogen reduction technologies.

Continuing our work,^25^ the present study focuses on the BSr_3_ superalkali cluster to elucidate its catalytic activity for ammonia synthesis under mild conditions. Using first-principles computations, three associative pathways (i.e., distal, alternating, and mixed) have been investigated. In these routes, the nitrogen atoms are progressively protonated until the ammonia molecules desorb from the BSr_3_ catalyst surface. The proposed associative mechanism for the dinitrogen fixation and protonation routes on the BSr_3_ superalkali cluster is a potential game-changer in the field of ammonia synthesis.

## 2 Computational Methods

Density functional theory (DFT) with the Pople split-valence basis set of triple-ζ quality [6–311++G­(3df,3pd)][Bibr ref31] was used to optimize the geometries of the BSr_3_–NH_*y*_NH_*x*_ (x, y = 0–3) species. The basis sets of Ahlrichs and co-workers, including the split valence and quadrupole zeta quality (QZVP) functions and Stuttgart-Dresden effective core potentials (ECP-28),^32^ were employed for strontium atoms (Def2QZVP).^33,34^ The Perdew–Burke–Ernzerhof (PBE0)^35^ exchange–correlation hybrid functional with dispersion correction by Grimme et al., with Becke–Johnson damping (PBE0-D3), was chosen as the DFT approach.^36^ For the PBE0-D3/6–311++G­(3df,3pd)+Def2QZVP relaxed structures, the vibrational frequencies were obtained at the same theory level. The post-Hartree–Fock method, the coupled-cluster method with single, double, and noniterative triple excitations, CCSD­(T), with the 6–311++G­(3df,3pd)+Def2QZVP basis, was employed to estimate the final energies of the species at their PBE0-D3/6–311++G­(3df,3pd)+Def2QZVP equilibrium geometries. Gaussian 16 (Rev. C.01) software was utilized for all computations.^37^ The CCSD­(T)/6–311++G­(3df,3pd)+Def2QZVP theory level was used to estimate the adsorption energy (*E*_ads_, eq 1) of the superalkali–N_2_ complex, hydrogenation energies (*E*_hyd_, eq 2) of BSr_3_–NH_*y*_NH_*x*_ species, and ammonia desorption energy (*E*_des_, eq 3).

1 $$E_{ads}=E_{{BSr}_{3}/N_{2}}-E_{{BSr}_{3}}-E_{N_{2}}$$

2 $$E_{hyd}=E_{{BSr}_{3}/N_{2}H_{\left(y+x\right)}}-E_{{BSr}_{3}/N_{2}H_{\left(y+x-1\right)}}-E_{H}-E_{e}$$

3 $$E_{des}=E_{{BSr}_{3}/{NH}_{\left(y+x-3\right)}}+E_{{NH}_{3}}-E_{{BSr}_{3}/N_{2}H_{\left(y+x\right)}}$$

The model of metallocene [CoCp*_2_, Cp* = η^5^-C_5_(CH_3_)_5_] and lutidinium [2,6-LutH]^+^ (Lut = 2,6-dimethylpyridine) can be considered as the source of the electron and proton, respectively. In this study, the electron affinity (E_e_) corresponds to the ionization energy for [CoCp*_2_] of −4.64 eV^26^ and the proton affinity energy (E_H_) required to abstract a proton from [2,6-LutH]^+^ was taken as −10.00 eV.^26^ The free-energy change (ΔG) of each hydrogenation step, at electrode potential U of 0 V and pH equal to zero, was estimated according to eq 4:

4 $$\Delta G=\Delta E+{\Delta E}_{ZPE}-T\Delta S$$

where Δ*E* is the reaction energy obtained from the CCSD­(T) theory level, Δ*E*_ZPE_ is the change of zero-point energy, *T* is the temperature (298.15 K), and Δ*S* refers to the entropy change. The zero-point energies were obtained from the vibrational frequencies, while entropies were estimated from vibrational frequencies, masses, and rotational constants derived from the optimized geometries.

## 3 Results

The following three-step reaction pathway for the N_2_ conversion into NH_3_ has been considered: (i) an adsorption of N_2_ on the BSr_3_ superalkali cluster, (ii) the triple N≡N bond activation via electron transfer from superalkali to the N_2_ molecule, and (iii) stepwise hydrogenation and reduction.

### 3.1 Adsorption of N_2_ on BSr_3_ Superalkali

The adsorption of a dinitrogen molecule on the catalyst surface is a prerequisite step for converting dinitrogen to form ammonia in a gas phase.^25,26,38^ Specifically, the initial structure of N_2_-adsorbed superalkali governs the subsequent reaction pathway for the reduction of dinitrogen to ammonia. Thus, different initial structures of BSr_3_/N_2_ were recalculated^25^ by combining molecular nitrogen with the vertex, edge, and face of the pyramidal-like structure of the BSr_3_ cluster via each possible orientation. The resulting PBE0-D3/6–311+G­(3df)+Def2QZVP equilibrium geometries of five low-lying BSr_3_–N_2_ isomers and the corresponding bond lengths and adsorption energies (*E*_ads_) for these complexes are given in Figure 1. The relative energies (with respect to the **1** BSr_3_–N_2_ ground state system, whose energy was taken as zero) were estimated to be in the range from 0.01 to 1.56 eV, implying that the **1**–**2** BSr_3_–N_2_ isomers can be formed in the gas phase. In the PBE0/6–311 + G­(3df)+Def2QZVP **1** BSr_3_–N_2_ global minimum structure, the B atom binds with three Sr atoms forming a pyramidal geometry with the dihedral B–Sr_1_–Sr_2_–Sr_3_ angle of 51°, in resemblance to the BSr_3_^+^ cation [ω­(BSr_1_Sr_2_Sr_3_) of 43° at the same theory level]. The B–Sr and Sr–Sr bonds are in the 2.628–2.964 Å and 3.878–3.894 Å ranges, respectively, resembling typical B–Sr and Sr–Sr bonds.^25,39^ These findings are consistent with the MP2­(full)/6–311+(3df)+Def2QZVP study on the BSr_3_–N_2_ system,^25^ which validates the accuracy of the present theoretical level.

**Figure 1:**
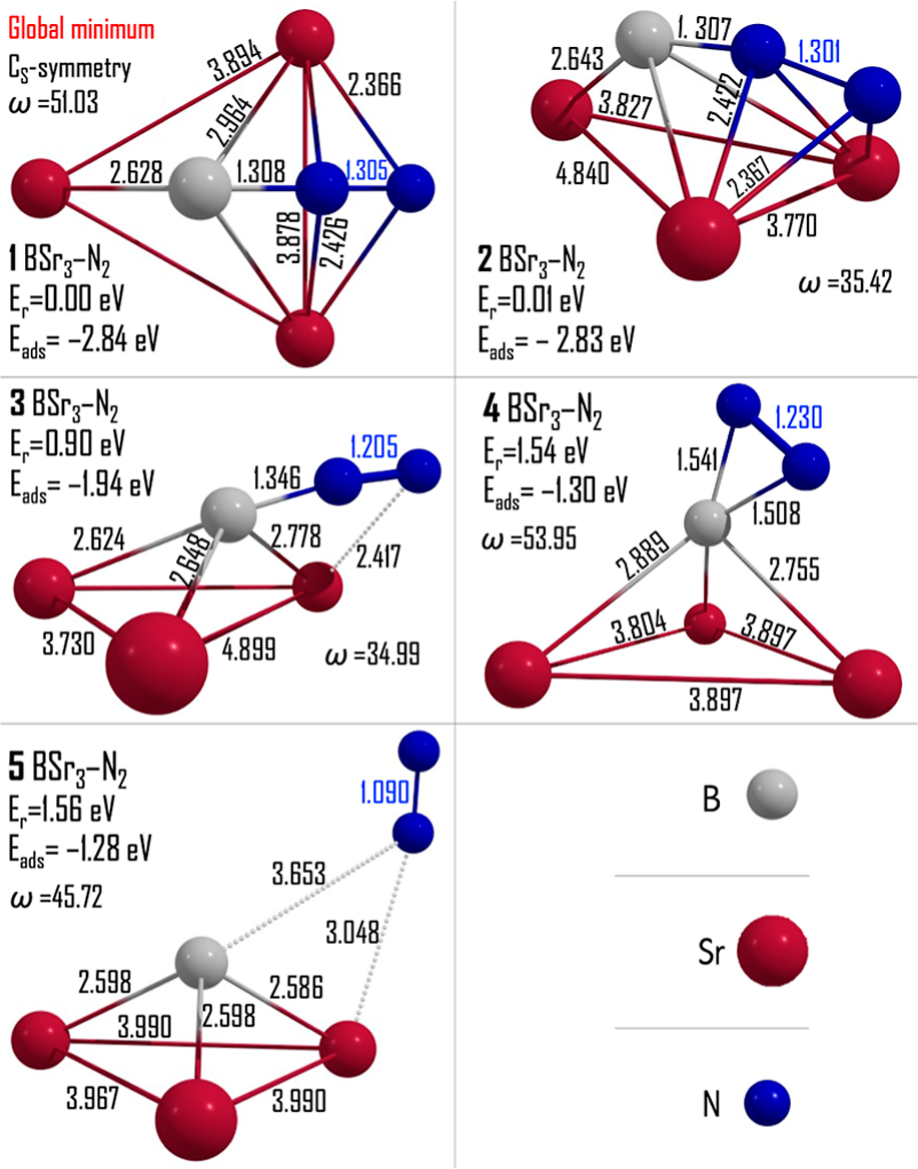
The PBE0-D3/6–311+G­(3df)+Def2QZVP equilibrium geometries of the BSr_3_–N_2_ system. The relative energies (E_r_) are obtained with respect to the lowest energy **1** BSr_3_–N_2_ isomer, whose energy was taken as zero. Bond lengths in Å and dihedral B–Sr_1_–Sr_2_–Sr_3_ angles (ω) in degrees. The zero-point vibrational corrected CCSD­(T,full)/6–311+G­(3df)+Def2QZVP//PBE0-D3/6–311+G­(3df)+Def2QZVP adsorption energy (E_ads_, in eV).

In the **1** BSr_3_–N_2_ ground state (Figure 1), the N_2_ subunit is attached to the boron atom via a side-on pattern and links to strontium atoms through four Sr–N bonds. Consequently, the BSr_3_ superalkali and dinitrogen counterparts are tightly combined with a significant adsorption energy of −2.84 eV, which is much larger than the dinitrogen adsorption energies of −0.06 eV on molybdenum boride (Mo_2_B),^40^ −0.45 to −1.60 eV on boron nanosheets,^41^ −2.05 eV on boron-doped C_2_N (B/C_2_N),^42^ and −2.09 eV on Ca_3_B superatom.^26^ Thus, molecular nitrogen can adsorb on the BSr_3_ superalkali at low temperatures. Furthermore, the N–N bond in the **1** BSr_3_–N_2_ ground state is 1.305 Å (Figure 1), which is considerably longer than that of (1.10 Å) the isolated dinitrogen molecule.^25,39^ This implies that the adsorbed N_2_ molecule on the BSr_3_ cluster has been significantly activated in the resulting **1** BSr_3_–N_2_ complex.

### 3.2 Triple N≡N Bond Activation via Electron Transfer from Superalkali to the N_2_ Molecule

The mechanism of dinitrogen adsorption on the BSr_3_ superalkali has been recently described by us elsewhere.^25^ It has been demonstrated that the BSr_3_/N_2_ system undergoes a significant decrease in energy as the fragments come together to form the BSr_3_–N_2_ ground state, as shown in Figure 2. However, upon approaching each other, the system temporarily stabilizes at a metastable intermediate, which is higher in energy (by 0.36 eV) than the isolated fragments.^25^ This energy uphill corresponds to an activation barrier associated with charge transfer from the superatomic cluster to the antibonding orbital of dinitrogen. This electronic rearrangement results in a significant elongation of the N–N bond (to 1.226 Å) and an increase in the total energy. As the reaction progresses, the system continues to evolve, eventually reaching the most energetically favorable state, which is 2.19 eV lower in energy than that of the isolated fragments. The strong superalkali–N_2_ interaction stabilizes the complex (by 2.19 eV) and is accompanied by substantial N–N distance elongation (to 1.308 Å), indicating pronounced activation of molecular nitrogen. These observations suggest that although BSr_3_ can efficiently bind N_2_, the process is not barrierless and requires a finite activation energy (of 0.36 eV) before the formation of the stable BSr_3_–N_2_ complex.

**Figure 2:**
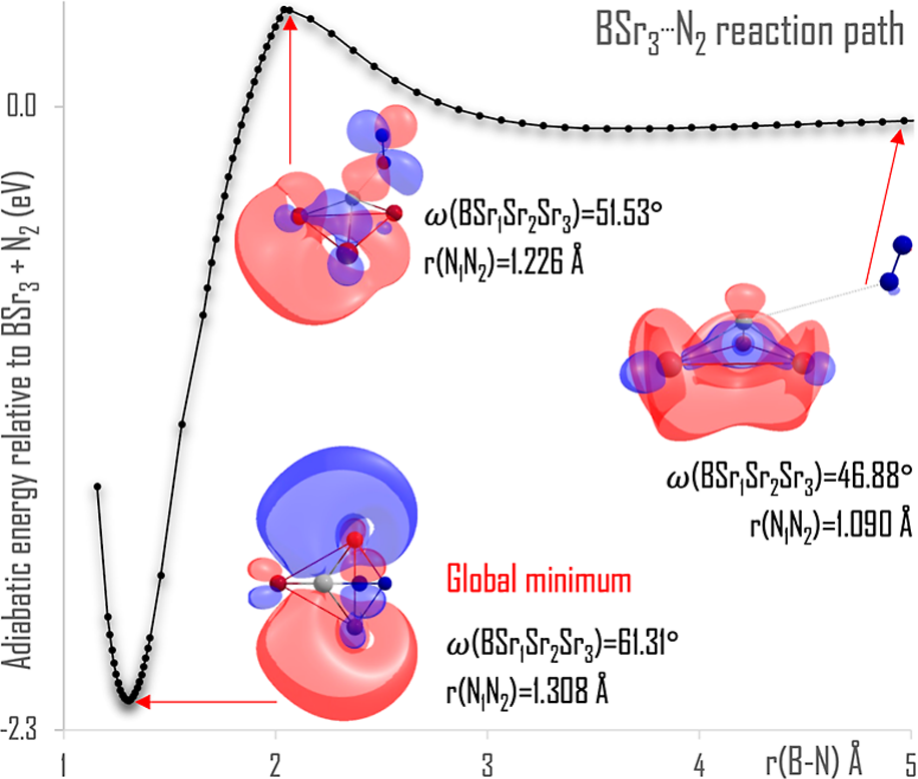
The adiabatic energy profiles for the formation of the BSr_3_–N_2_ complex according to the BSr_3_ + N_2_ → BSr_3_–N_2_ reaction. The total energy was corrected for basis set superposition error (BSSE) using the counterpoise method of Boys and Bernardi, treating the BSr_3_/N_2_ system as two interacting fragments (i.e., BSr_3_ and N_2_).^25,43,44^ The relative energies are obtained in relevance to the sum of energies of isolated fragments [BSr_3_; N_2_]. The single occupied molecular orbitals (SOMOs) holding the excess electron are shown for the equilibrium geometries corresponding to r = 1.302 Å (global minimum), r = 1.965 Å (local minimum), and r = 4.965 Å. Reproduced from ref 25 with permission from the Royal Society of Chemistry.

### 3.3 Hydrogenation of Nitrogen Atoms on N_2_-Adsorbed Superalkali

Since the N_2_ adsorption energy (*E*_ads_ = −2.84 eV) of the most stable **1** BSr_3_–N_2_ complex (Figure 1) is larger than that on Mo_2_N (−0.06 eV), boron nanosheets (−1.60 eV), and sp^2^-hybridized B/C_2_N (−2.05 eV), the BSr_3_ superalkali can be regarded as an effective agent for N_2_ activation.^40–42^ The activated N_2_ molecule can be further converted to ammonia through a stepwise hydrogenation process. The hydrogenation of nitrogen atoms on the N_2_-adsorbed superalkali surface determines the reaction pathways for converting N_2_ into NH_3_ in the gas phase. Here, we investigated associative mechanisms in which adsorbed N_2_ molecules are gradually reduced to form ammonia. These associative mechanisms follow three possible pathways that differ in the protonation route and order of each ammonia molecule release, as depicted in Section 3.4. However, their common characteristic is the gradual cleavage of the triple bond by progressive hydrogenation to the NH_3_ molecule.

Different initial structures of hydrogenated BSr_3_–N_2_ species were constructed by combining the hydrogen atom with the first (N^B^) and second (N^Sr^) nitrogen atoms of the lowest-energy **1** BSr_3_–N_2_ complex via all possible combinations. The optimized geometric structures of low-lying BSr_3_–NH_*y*_NH_*x*_ (x, y = 0–3) isomers and the corresponding Sr–N, B–N, N–H bond lengths are given in Figure 3, while the N–N distances and hydrogenation energies for these complexes Figure 4 are shown in Figure 5 and Figure 6. The geometrical structures shown in Figure 3 are significant in understanding the nitrogen hydrogenation mechanisms on a superalkali surface, which will be discussed in the following section.

**Figure 3:**
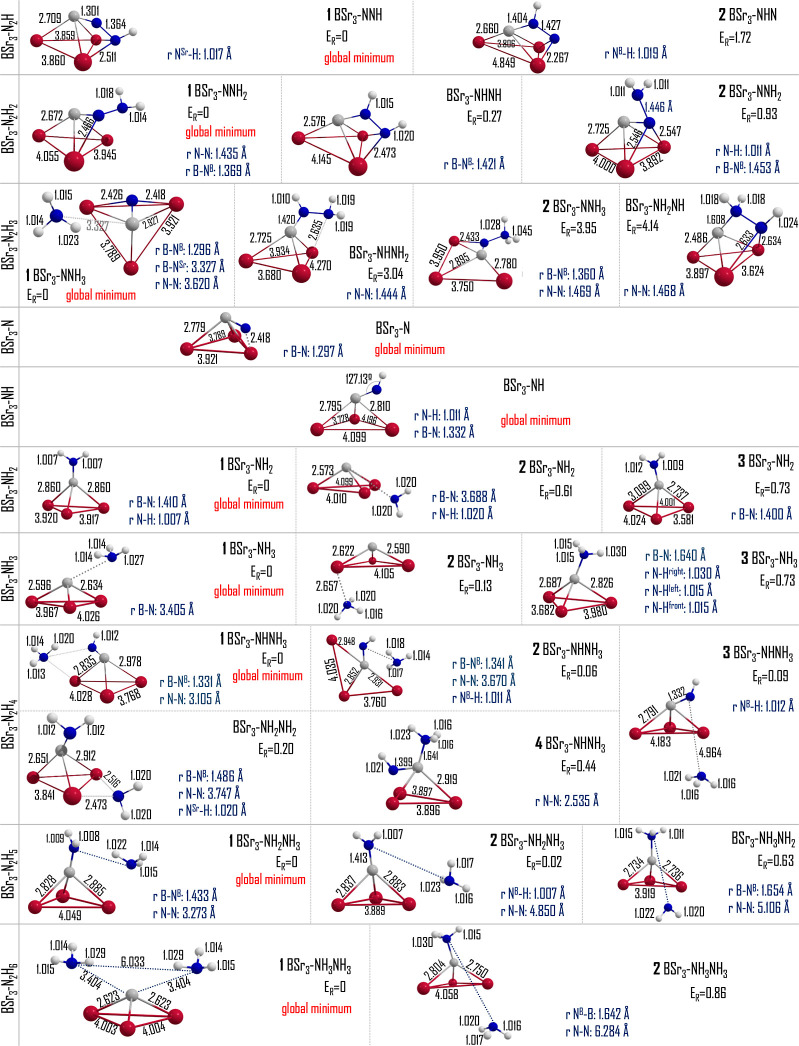
The PBE0-D3 relaxed atomic configurations of the BSr_3_–*NH*_*y*_ *NH*_*x*_ (x, y = 0–3) intermediates of each step for N_2_ fixation on the BSr_3_ cluster. Dotted lines separate constitutional isomers, and their relative energies (in eV) are obtained in relation to the corresponding global minima (indicated in red). Bond lengths are given in Å. See Table S1 in the Supporting Information for spin states of the relaxed structures.

**Figure 4:**
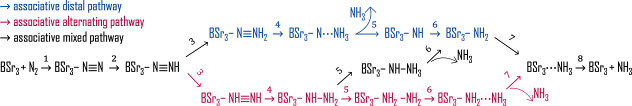
Schematic depiction of three reaction mechanisms for the nitrogen electroreduction reaction (NRR) to ammonia on the BSr_3_ cluster.

**Figure 5:**
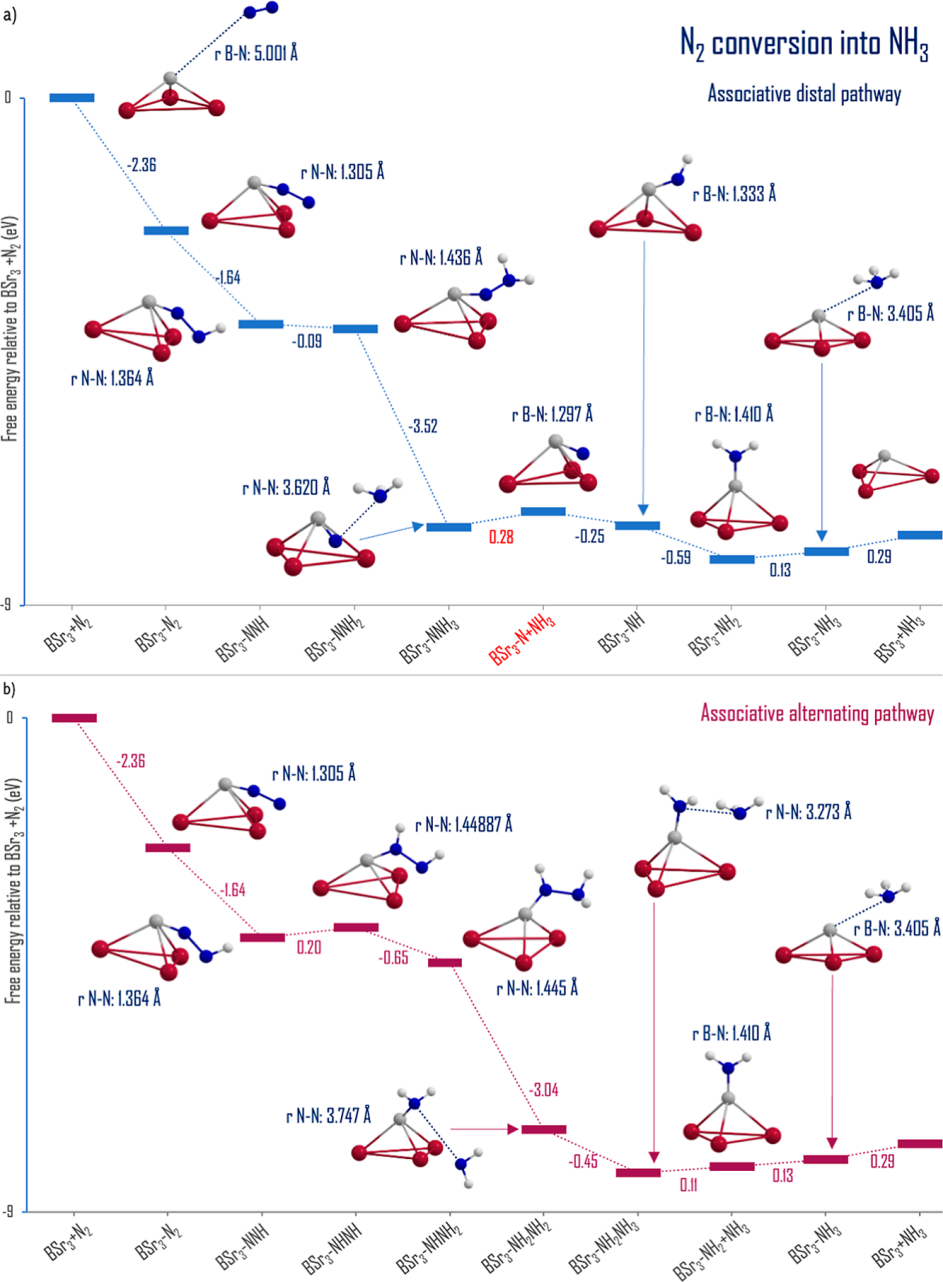
The reaction mechanisms for the nitrogen electroreduction reaction (NRR) to ammonia on the BSr_3_ cluster catalyst: (a) associative distal pathway and (b) associative alternating pathway. The relative free energies are obtained in relevance to the sum of energies of isolated fragments [BSr_3_; N_2_]. For each reaction step, the free-energy change (ΔG, in eV) is also provided. For the distal pathway, the ΔG for the desorption of the first NH_3_ molecule is indicated by the positive value in the reaction pathway (marked in red).

**Figure 6:**
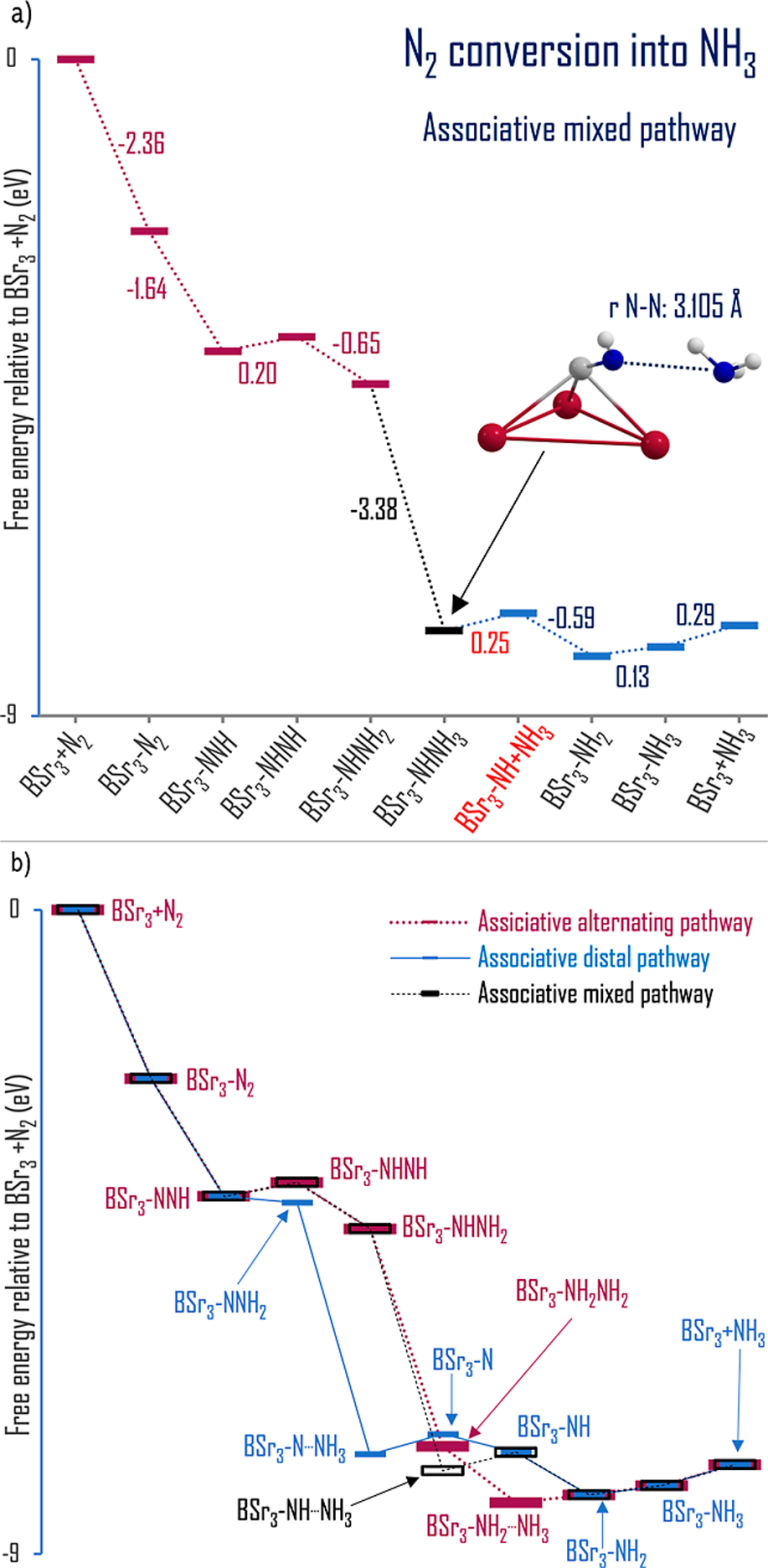
The reaction mechanisms for the NRR to ammonia on the BSr_3_ cluster catalyst: (a) associative mixed pathway and (b) combined energy profile for associative alternating (in purple), distal (in blue), and mixed (in black) pathways. The relative free energies are obtained in relevance to the sum of energies of isolated fragments [BSr_3_; N_2_]. For the mixed pathway, the free-energy change (ΔG, in eV) of each reaction step is depicted, while the ΔG for the first ammonia desorption is indicated by the positive value in the reaction pathway (marked in red).

### 3.4 Mechanism of NH_3_ Formation by the Reduction of N_2_ Adsorbed on BSr_3_ Superalkali

The electrochemical nitrogen reduction reaction can occur through different pathways depending on the catalyst used.^45,46^ The mechanism proposed for the most common ammonia synthesis method, such as the Haber–Bosch method, is postulated to proceed via a dissociative pathway. The dissociative path primarily follows the Langmuir–Hinshelwood mechanism and requires extreme conditions to overcome the kinetic barriers that prevent the cleavage of the triple N_2_ bond before hydrogenation begins.^47^ In contrast, under mild conditions, the reaction proceeds via an associative mechanism in which adsorbed N_2_ molecules are reduced stepwise. In the associative route, the triple bond weakens during hydrogenation, and the nitrogen atoms are progressively protonated until the ammonia molecules desorb from the catalyst surface. Six net coupled proton and electron-transfer steps are involved in the nitrogen reduction process (N_2_ + 6H^+^ + 6e^–^ → 2NH_3_). In line with previous theoretical studies,^26^ for simplicity, the model of metallocene [CoCp*_2_, Cp* = η^5^-C_5_(CH_3_)_5_] and lutidinium [2,6-LutH]^+^ (Lut = 2,6-dimethylpyridine) is assumed as the sources of electrons and protons, respectively. Each coupled proton–electron-transfer step involves the transfer of a proton–electron pair from the solution to an adsorbed species on the catalyst surface.

We investigated three associative mechanisms for the reduction of nitrogen to ammonia on the BSr_3_ superalkali cluster, as illustrated in Figure 4. These mechanisms involve the stepwise coupling of proton and electron transfers, with each step contributing to the gradual reduction of nitrogen to ammonia.

#### 3.4.1 Associative Distal Pathway for N_2_ Conversion into NH_3_ Catalyzed by BSr_3_

The most probable mechanistic pathway is depicted in Figure 5a. It involves successive hydrogenation at one nitrogen atom (N^Sr^ coordinated to Sr atoms) until the release of the first NH_3_ molecule, followed by the hydrogenation of the remaining nitrogen atom (N^B^ coordinated to a boron atom) to form the second ammonia molecule. The first hydrogenation step on the **1** BSr_3_–N_2_ complex exhibits a negative free-energy change of −1.64 eV, indicating that the initial hydrogenation drives the subsequent nitrogen reduction process. Upon the first hydrogenation, the N–N bond in the **1** BSr_3_–NNH ground state (Figure 3) elongates to 1.364 Å, which is considerably longer than that of (1.305 Å) the **1** BSr_3_–N_2_ complex (Figure 1). As shown in Figure 5a, after the first hydrogenation of the nitrogen atom, the generated **1** BSr_3_–NNH undergoes successive hydrogenation to form **1** BSr_3_–NNH_2_ (Figure 3), accompanied by an energy release of −0.09 eV. This step elongated the N–N distance from 1.364 to 1.436 Å (Figure 5a). Similarly, the formation of BSr_3_–NNH_3_ is energetically preferred in the third hydrogenation step. Specifically, the formation of **1** BSr_3_–NNH_3_ (Figure 3) releases an energy of −3.52 eV (Figure 5a). Moreover, the N–N distance in **1** BSr_3_–NNH_3_ has been elongated to 3.620 Å, which is conducive to the release of the first NH_3_ molecule. The first NH_3_ molecule desorbs from the BSr_3_ cluster, absorbing a small amount of energy (+0.28 eV). The remaining BSr_3_–N species (Figure 3) undergoes a sequence of hydrogenation steps to gradually generate the BSr_3_–NH, **1** BSr_3_–NH_2_, and **1** BSr_3_–NH_3_ species (Figure 3), with free-energy changes of −0.25, −0.59, and +0.13 eV, respectively (Figure 5a). Finally, the second NH_3_ molecule desorbs from the BSr_3_ superalkali by absorbing the energy of +0.29 eV (Figure 5a), and thus, the BSr_3_ is regenerated for the next catalytic cycle of the nitrogen reduction reaction.

Due to the efficient activation of N_2_ by the BSr_3_ superalkali cluster, the formation of **1** BSr_3_–NNH, **1** BSr_3_–NNH_2_, and **1** BSr_3_–NNH_3_ intermediates is exergonic. The desorption of the first NH_3_ molecule becomes the potential-limiting step, characterized by absorbing a small energy of +0.28 eV. The preceding three exergonic hydrogenation steps can help offset the energy cost of ammonia desorption, ensuring overall thermodynamic favorability. Additionally, the free-energy change of the first NH_3_ desorption is lower than the 0.71–1.39 eV range observed for the potential-limiting step of the nitrogen reduction reaction on various reported catalysts.^26,48,49^ Therefore, the BSr_3_ superalkali cluster is a promising catalyst with exceptional performance in N_2_ reduction and ammonia synthesis.

#### 3.4.2 Associative Alternating Pathway for N_2_ Conversion into NH_3_ Catalyzed by BSr_3_

As shown in Figure 5b, the associative alternating mechanism involves six consecutive hydrogenation steps during which the triple N≡N bond is progressively weakened and ultimately cleaved, enabling ammonia desorption. In the alternating route, both nitrogen atoms adsorbed on the BSr_3_ superalkali are sequentially protonated. The **1** BSr_3_–N_2_ system undergoes a stepwise hydrogenation, gradually generating the **1** BSr_3_–NNH, BSr_3_–NHNH, BSr_3_–NHNH_2_, BSr_3_–NH_2_NH_2_, **1** BSr_3_–NH_2_NH_3_, and **1** BSr_3_–NH_3_ species (Figure 3), with corresponding free-energy changes of −1.64, +0.20, −0.65, −3.04, −0.45, and +0.13 eV, respectively (Figure 5b). In this associative alternating pathway, the protonation of **1** BSr_3_–NNH to form BSr_3_–NHNH represents the potential-limiting step due to its positive ΔG value (+0.20 eV, Figure 5b). The first NH_3_ molecule desorbs from the BSr_3_ cluster, absorbing a small amount of energy (+0.11 eV). The subsequent protonation of **1** BSr_3_–NH_2_ to form the **1** BSr_3_–NH_3_ intermediate elongates the B–N distance to 3.405 Å, facilitating the release of the second NH_3_ molecule (ΔG of +0.29 eV, Figure 5b). Although a preceding exergonic hydrogenation step partially offsets the energy demand of this protonation, the presence of four endergonic steps in the overall route increases the total energetic requirement, potentially leading to a higher overpotential or activation barrier for the transformation. Thus, the associative alternating route path is less energetically favorable than the associative distal pathway.

#### 3.4.3 Associative Mixed Route Path for N_2_ Conversion into NH_3_ Catalyzed by BSr_3_

In the mixed route depicted in Figure 6a, the exergonic hydrogenation (ΔG = −1.64 eV) begins at the nitrogen atom coordinated to strontium atoms (N^Sr^), followed by the endergonic hydrogenation (ΔG = +0.20 eV) at the nitrogen atom coordinated to a boron atom (N^B^) that results in the BSr_3_–NHNH intermediate. In the following steps, the two subsequent hydrogenations occur on the first nitrogen atom (N^Sr^) to form **1** as BSr_3_–NHNH_3_ species. In the **1** BSr_3_–NHNH_3_ intermediate (Figure 3), the N–N distance is elongated to 3.105 Å, making it vulnerable to the first NH_3_ desorption. Following the endergonic desorption of the first NH_3_ molecule (ΔG of +0.25 eV, Figure 6a) from the **1** BSr_3_–NHNH_3_ intermediate, the remaining nitrogen atom (N^B^) is further hydrogenated to synthesize the **1** BSr_3_–NH_3_ intermediate. Finally, the second ammonia molecule can be desorbed from the BSr_3_ cluster, overcoming a positive ΔG of +0.29 eV (Figure 6a).

In the mixed associative pathway, four elementary steps are endergonic: (i) the protonation of **1** BSr_3_–NNH to BSr_3_–NHNH intermediate (ΔG of +0.20 eV), (ii) the desorption of the first NH_3_ molecule (ΔG of +0.25 eV), (iii) the hydrogenation to **1** BSr_3_–NH_3_ intermediate (ΔG of +0.13 eV), and (iv) desorption of the second NH_3_ molecule (ΔG of +0.29 eV). A higher number of endergonic steps makes the overall reaction pathway energetically less favorable as multiple uphill transitions increase the total energy demand and lower the overall catalytic efficiency. Thus, the mixed mechanism is less energetically favorable than the associative distal mechanism, as shown in Figure 6b.

#### 3.4.4 Comparative Endergonic Reaction Step Analysis of the Associative NRR Pathways

To facilitate comparison of the three associative mechanisms, the endergonic step parameters extracted from the free-energy profiles in Figures 5 and 6 are summarized in Table 1. The analysis identifies the distal pathway as the most favorable route for ammonia formation on the BSr_3_ surface. In the distal mechanism (indicated in blue in Figure 6b), protonation proceeds preferentially at the nitrogen atom distal to the cluster, leading to a smooth and energetically gradual hydrogenation sequence. The potential-determining step corresponds to the desorption of the first NH_3_ molecule, with ΔG = 0.28 eV. Notably, all preceding proton-coupled electron-transfer steps remain thermodynamically accessible, indicating efficient stabilization of hydrogenated intermediates and effective charge redistribution from the electron-rich BSr_3_ core into the N_2_-derived species. The final (sixth) protonation and subsequent structural rearrangement absorb a small amount of energy (+0.13 eV) before the second ammonia molecule desorbs (ΔG = 0.29 eV).

**Table 1:** Comparative Endergonic Reaction Step Analysis of Associative NRR Pathways on BSr_3_ Superalkali^a^

associative pathway	number of endergonic steps	endergonic step description	endergonic step	ΔG (eV)
distal	3	first NH_3_ desorption	BSr_3_–NNH_3_ → BSr_3_–N + NH_3_	+0.28
		sixth protonation	BSr_3_–NH_2_ + H^+^ + e^–^ → BSr_3_–NH_3_	+0.13
		second NH_3_ desorption	BSr_3_–NH_3_ → BSr_3_ + NH_3_	+0.29
alternating	4	second protonation	BSr_3_–NNH + H^+^ + e^–^ → BSr_3_–NHNH	+0.20
		first NH_3_ desorption	BSr_3_–NH_2_NH_3_ → BSr_3_–NH_2_ + NH_3_	+0.11
		sixth protonation	BSr_3_–NH_2_ + H^+^ + e^–^ → BSr_3_–NH_3_	+0.13
		second NH_3_ desorption	BSr_3_–NH_3_ → BSr_3_ + NH_3_	+0.29
mixed	4	second protonation	BSr_3_–NNH + H^+^ + e^–^ → BSr_3_–NHNH	+0.20
		first NH_3_ desorption	BSr_3_–NHNH_3_ → BSr_3_–NH + NH_3_	+0.25
		sixth protonation	BSr_3_–NH_2_ + H^+^ + e^–^ → BSr_3_–NH_3_	+0.13
		second NH_3_ desorption	BSr_3_–NH_3_ → BSr_3_ + NH_3_	+0.29

a For each endergonic reaction step, the free-energy change (ΔG, in eV) is provided.

In contrast to the distal route, both the alternating and the mixed pathways exhibit an additional endergonic step associated with hydrogenation. The alternating addition of hydrogen atoms to both nitrogen centers (BSr_3_–NNH + H^+^ + e^–^ → BSr_3_–NHNH, ΔG = 0.20 eV) results in less efficient electronic delocalization and an enhanced energetic demand than the distal route. Overall, the comparative free-energy analysis of the three associative pathways demonstrates that BSr_3_ preferentially promotes the distal associative mechanism. The relatively low free energy of the potential-determining step (ΔG = 0.28 eV) confirms that the BSr_3_ cluster provides sufficient electron density to activate N_2_ while avoiding excessive stabilization that would hinder product release. The balance between strong initial adsorption and controlled intermediate stabilization underpins the catalytic competence of the BSr_3_ cluster for ambient nitrogen reduction.

### 3.5 Associative Distal Route Path for Efficient Nitrogen Reduction into Ammonia

The following three criteria should be passed for an eligible electrocatalyst for the nitrogen reduction reaction (NRR): (i) the catalyst can facilitate the chemisorption of the N_2_ molecule, thereby enabling the effective activation of its inert N≡N triple bond, (ii) the catalyst might selectively stabilize N_2_H* intermediate, and (iii) the catalyst might destabilize NH_2_* species and facilitate the reduction of the overpotential.^50^ Following the above first criterion, the obtained ΔG_*ads*_ value of N_2_ adsorption on the BSr_3_ cluster is negative (−2.36 eV), indicating that this superalkali is sufficient as a nitrogen reduction reaction electrocatalyst due to its efficient performance in N_2_ activation. Regarding the second criterion, the BSr_3_ superalkali satisfies this requirement as it exhibits sufficient stabilization toward the N_2_H* intermediate (ΔG_*ads*_ of −4.40 eV). Regarding the third requirement, NH_2_* is adsorbed with a free-energy change of −4.36 eV, indicating a slightly weaker interaction than that of N_2_H*. Although the numerical difference between these adsorption energies is small and within the typical uncertainty of DFT calculations, the overall trend qualitatively satisfies the established screening criteria. Thus, the BSr_3_ superalkali can be considered to be a promising catalyst for the nitrogen reduction reaction.

To gain a deeper insight into the catalytic performance of the BSr_3_ cluster, Figure 7 compares the free-energy change associated with the potential-determining step (PDS) for electrochemical NH_3_ formation catalyzed by BSr_3_ superalkali with that of recently reported catalysts.^51–55^ As shown in Figure 5a, NH_3_ synthesis on the BSr_3_ proceeds via an associative distal mechanism, where the desorption of the first NH_3_ molecule constitutes the PDS with a free-energy change of only 0.28 eV. This free-energy change of PDS is lower than that recently reported for transition-metal carbides with carbon vacancies, such as VC (0.58 eV) and WC (0.35 eV) surfaces, where the PDS involves NH_2_ hydrogenation and surface carbon protonation, respectively.^52,53^ Even larger free-energy changes associated with PDSs are observed for the (110) facets of rock salt structures of tantalum carbide (TaC) and tungsten carbide (WC), at 0.66 and 0.82 eV, respectively.^51^ Although single-atom catalysts (SACs) have demonstrated improved N_2_ activation, their limiting steps generally remain above the PDS’s free-energy change of BSr_3_ superalkali. For example, Os atoms loaded on the two-dimensional AlN monolayer with Al monovacancy (Os@AlN) exhibit a limiting potential step of 0.46 eV along the NRR distal pathway.^54^ Similarly, the estimated PDS free-energy change of NRR on the BSr_3_ cluster is also lower than that for the NRR catalyzed by single Au or Fe atoms supported on graphitic C_2_N (Fe–C_2_N: 0.7 eV and Au–C_2_N: 1.6 eV).^55^ The exceptionally small free-energy change associated with PDS observed for BSr_3_ underscores the intrinsic thermodynamic advantage of N_2_ reduction, highlighting its potential as an efficient catalyst for sustainable NH_3_ production.

**Figure 7:**
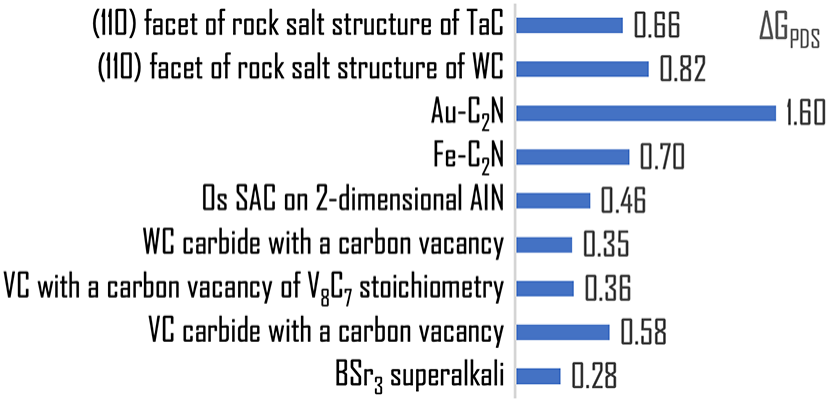
Comparison of the potential-determining step (PDS) free-energy changes (ΔG_PDS_ in eV) for electrochemical NH_3_ formation on different catalysts. Literature data are taken from [51–55].

### 3.6 Catalytic Potential of the BSr_3_ Superatom: Comparison with Small Metal Clusters Used in Catalysis

The BSr_3_ superatom, owing to its discrete electronic structure and low ionization energy, can be advantageous in specific catalytic applications. Small metal clusters, including those composed of platinum, have recently been shown to catalyze multistep reactions.^56,57^ For instance, fully exposed platinum clusters comprising an average of four platinum atoms supported on nanodiamond@graphene demonstrated excellent catalytic performance, underscoring the efficiency of small clusters in catalyzing complex reactions.^41^ Extended catalytic surfaces, such as metal oxides and transition metals, stabilize reaction intermediates through delocalized electronic band states and multisite coordination across a periodic lattice.^58^ While such delocalization enables strong adsorption and charge redistribution, it often gives rise to scaling relationships that limit independent tuning of elementary steps. By contrast, small molecular clusters, such as the BSr_3_ superalkali, have discrete frontier orbitals and a well-defined coordination environment. Electronic perturbations are spatially confined within a finite cluster framework, allowing targeted charge transfer into specific adsorbate orbitals rather than collective redistribution across an extended surface.^25^

The BSr_3_ superalkali cluster can donate electron density to molecular nitrogen, a crucial step in the electrochemical reduction of nitrogen to ammonia. The small size of molecular clusters creates a high density of active sites, facilitating weakening of the triple N≡N bond and making it easier for subsequent hydrogenation steps to occur. Computational studies of supported atomic clusters have shown that an Mo_2_@C_2_N and Mn_2_–C_2_N can serve as heterogeneous catalysts for the adsorption and activation of substrates (such as molecular nitrogen).^45,59,60^ The size of the molecular clusters determines the fraction of surface atoms, significantly affecting both catalytic activity and selectivity. In supported atomic clusters, most metal atoms are exposed as much as possible and are available to reactant molecules. Therefore, supported atomic clusters exhibit significantly higher atom utilization efficiency in catalytic reactions than corresponding nanoparticles or single-atom catalysts.^61^ Experimental studies have further demonstrated that small clusters embedded within metal–organic frameworks (MOFs) can retain catalytic activity while benefiting from spatial confinement and structural stabilization.^62^ Tiburcio et al. demonstrated that the Ag_2_ clusters embedded within an MOF [with a resulting [Ag_2_]@Ag_2_Na_2_[Ni_4_[Cu_2_(*N*,*N′*-2,4,6-trimethyl-1,3-phenylenebis­(oxamate))_2_]_3_]·48H_2_O formula] can be used as efficient and recoverable catalysts for heterogeneous catalysis in organic synthesis.^63^ These studies suggest that although small clusters may offer fewer adsorption sites than extended surfaces, their discrete electronic structure can enable selective activation pathways that circumvent conventional scaling constraints.

Translating the BSr_3_ cluster into a heterogeneous catalyst will require a judicious support selection to stabilize the cluster while preserving its superalkali character. Two-dimensional carbon-based supports offer tunable anchoring sites and electronic coupling, enabling localized charge transfer and mitigating aggregation.^64,65^ For example, nitrogen-doped graphene and graphene oxide structures create Lewis basic sites that may preferentially interact with the electron-deficient boron atom of the BSr_3_ cluster, inducing charge transfer that enriches the π* antibonding orbital of coordinated N_2_ and thereby weakening the N–N bond.^66^ Complementary, embedding BSr_3_ within MOF cavities (such as zirconium-based UiO-66 with the Zr_6_O_4_(OH)_4_(OOC–C_6_H_4_–COO)_6_ formula)^67^ may enhance stability through framework functionalization. Charge redistribution at the cluster–support interface can fine-tune frontier orbital alignment, strengthening charge donation into N_2_ antibonding orbitals without extensive delocalization across an extended lattice. Collectively, support–cluster interactions might not only anchor the BSr_3_ cluster but also offer pathways to enhance charge transfer, adjust electronic structure, and alter activation barriers, while maintaining the intrinsic discrete features of the superalkali cluster.

From a synthetic perspective, supported BSr_3_ catalysts can be prepared via wet-chemical immobilization of preformed clusters onto functionalized graphene or MOF surfaces, via atomic layer deposition (ALD) to achieve uniform cluster coverage, or via gas-phase cluster beam deposition for precise control over cluster size.^68^ For instance, a synthetic route to a BSr_3_ cluster anchored to a MOF can proceed via in situ cluster generation during MOF crystallization, in which boron and strontium precursor compounds (e.g., borate esters, strontium alkoxides) are introduced during synthesis and subsequently reduced under controlled conditions (mild hydrogenation, NaBH_4_ addition). Structural and electronic characterization of supported BSr_3_ cluster will benefit from a combination of high-resolution techniques such as (i) aberration-corrected scanning transmission electron microscopy (AC-STEM) to directly image cluster size and distribution; (ii) X-ray absorption spectroscopy (XAS) to probe local coordination environment and oxidation states; and (iii) and in situ or operando vibrational spectroscopies (including Raman and infrared with probe molecules such as CO) to track BSr_3_ cluster and BSr_3_–NH_*y*_NH_*x*_ intermediates behavior under reaction conditions.^60^ Operando techniques under reaction conditions will be critical for linking the cluster structure to catalytic function.

Overall, the BSr_3_ superatom is a promising candidate for catalytic nitrogen reduction. Its small size and electron-donating ability are parallel examples of metal cluster catalysis. Stabilization of N_2_ and its hydrogenated derivatives occurs through localized electron donation into the π* antibonding orbitals, accompanied by directional B–N and B–Ae bridging interactions. The resulting charge transfer is spatially confined within the cluster framework rather than distributed over an extended lattice, providing selective and controllable intermediate binding. While further computational and experimental studies are required to assess the long-term stability and scalability, the BSr_3_ cluster offers a blueprint for noble-metal-free catalysts for multielectron transformations such as nitrogen reduction.

## 4 Summary

Based on first-principles calculations, the BSr_3_ superalkali system has been shown to act as an efficient electron donor for dinitrogen activation and to represent a promising noble-metal-free catalyst for electrochemical ammonia synthesis. The nitrogen reduction reaction (NRR) on BSr_3_ proceeds through three key stages: (i) N_2_ adsorption on the BSr_3_ cluster, (ii) triple N≡N bond activation via electron transfer from superalkali to the N_2_ molecule, and (iii) stepwise protonation and reduction leading to NH_3_ formation. It has been demonstrated that BSr_3_ superalkali donates electron density to the N_2_ molecule, forming a superalkali–N_2_ complex. This electron donation weakens a N≡N triple bond via partial population of its antibonding orbitals. As the electron density is transferred to the N_2_ molecule from the BSr_3_ superalkali, the bond order of the N≡N triple bond decreases, making the molecule more reactive. This electronic activation is a crucial process that enables subsequent chemical transformations. In a catalytic setup, the activated N_2_ undergoes stepwise addition of protons (H^+^) and additional electrons, resulting in intermediates such as BSr_3_–NNH, BSr_3_–NNH_2_, BSr_3_–NNH_3_, BSr_3_–NH, and BSr_3_–NH_2_, ultimately forming ammonia molecules. Each subsequent hydrogenation step is accompanied by further elongation of the N–N bond, directly reflecting progressive weakening of the N≡N triple bond and stepwise activation of N_2_. The N–N bond length is consecutively stretched from 1.10 Å in a free N_2_ molecule to 1.305 Å in BSr_3_–N_2_ through 1.364 Å in BSr_3_–NNH and 1.436 Å in BSr_3_–NNH_2_, ultimately reaching 3.620 Å in BSr_3_–N···NH_3_, where the N–N bond cleavage occurs. This continuous bond elongation provides a structural descriptor of catalytic activation and confirms the feasibility of N_2_ reduction on the BSr_3_ cluster.

Mechanistically, the most favorable pathway follows the associative distal route. It involves successive hydrogenation at one nitrogen atom (N^Sr^, coordinated to the Sr atoms) until the release of the first ammonia molecule, followed by the hydrogenation of the remaining nitrogen atom (N^B^, bound to a boron atom of the BSr_3_ superalkali) to form the second NH_3_ molecule. The free-energy analysis identifies NH_3_ desorption as the potential-determining step, while preceding hydrogenation steps are exergonic and partially offset the energetic demand of product release.

Overall, BSr_3_ enables efficient N_2_ activation through strong electronic coupling and progressive N≡N bond weakening, highlighting superalkali clusters as a new class of electron-rich catalytic motifs for sustainable ammonia synthesis. These findings establish a structure–properties relationship between electron transfer, bond elongation, and catalytic performance, and may guide the rational design of superalkali-based systems for nitrogen fixation.

## Supplementary Material



## Data Availability

The data supporting this article have been deposited in the repository (DOI: 10.18150/KUWQNU).

## Notes

Spin states of BSr_3_–NH_*y*_NH_*x*_ (*x*, *y* = 0–3) species and molecular coordinates (PDF)
