# Supplementary material for: BSr3 Superalkali as a Promising Catalyst for Ambient Nitrogen Reduction: A Pathway toward Efficient Ammonia Synthesis
Source: Precis Chem. 2026 Mar 12;4(7):938–49. doi: 10.1021/prechem.5c00284 (PMC13417513; doi:10.1021/prechem.5c00284)
Supplement: Supplementary file 1 [file pc5c00284_si_001.pdf]

SUPPORTING INFORMATION

BSr_3 superalkali as a promising catalyst for ambient nitrogen reduction: a pathway toward efficient ammonia synthesis

Natalia Wiszowska[1], Natalia Rogoża[1], Celina Sikorska[1,2, *]

[1] Faculty of Chemistry, University of Gdańsk,
Fahrenheit Union of Universities in Gdańsk, Wita Stwosza 63, 80-308 Gdańsk, Poland

[2] Department of Physics, The University of Auckland
38 Princes Street, Auckland 1010, New Zealand

[*] Corresponding author: celina.sikorska@ug.edu.pl

1. Electronic spin in BSr_3–NH_yNH_x (x, y = 0–3) species

Table S 1

Spin states and eigenvalues of spin-squared operator [$<S^2>$] of the PBE0-D3/6-311+G(3df)+Def2QZVP equilibrium geometries of BSr_3–NH_yNH_x (x, y = 0–3) molecular systems. The CCSD(T,full)/6-311+G(3df)+Def2QZVP// PBE0-D3/6-311+G(3df)+Def2QZVP relative energies (E_r, in eV) are obtained in relation to the corresponding global minima (indicated in red in Figure 3 of the main text).

Molecular system	Constitutional isomers	E_r (eV)	Spin state	$<S^2>$
BSr_3-N_2	**1**-BSr_3-NN	0.00	dublet	0.7503
BSr_3-N_2H	**1** BSr_3-NNH	0.00	singlet	0
	**2** BSr_3-NHN	1.72	singlet	0
BSr_3-N_2H_2	**1** BSr_3-NNH_2	0.00	dublet	0.7698
	BSr_3-NHNH	0.27	dublet	0.8095
	**2** BSr_3-NNH_2	0.93	dublet	0.8250
BSr_3-N_2H_3	**1** BSr_3-NNH_3	0.00	singlet	0
	BSr_3-$NHNH_2$	3.04	singlet	0
	**2** BSr_3-NNH_3	3.95	singlet	0
	BSr_3-NH_2NH	4.14	singlet	0
BSr_3-N	**1** BSr_3-N	0.00	triplet	2.0001
	**2** BSr_3-N	0.04	singlet	0
BSr_3-NH	BSr_3-NH	0.00	dublet	0.7817
BSr_3-NH_2	**1** BSr_3-NH_2	0.00	singlet	0
	**2** BSr_3-NH_2	0.61	singlet	0
	**3** BSr_3-NH_2	0.73	triplet	2.0015
BSr_3-NH_3	**1** BSr_3-NH_3	0.00	dublet	0.7554
	**2** BSr_3-NH_3	0.13	dublet	0.7600
	**3** BSr_3-NH_3	0.73	dublet	0.7717
BSr_3-N_2H_4	**1** BSr_3-$NHNH_3$	0.00	dublet	0.7560
	**2** BSr_3-$NHNH_3$	0.06	dublet	0.7592
	**3** BSr_3-$NHNH_3$	0.09	dublet	0.7836
	BSr_3-NH_2NH_2	0.20	dublet	0.8705
	**4** BSr_3-$NHNH_3$	0.44	dublet	0.7911
BSr_3-N_2H_5	**1** BSr3-NH_2NH_3	0.00	singlet	0
	**2** BSr_3-NH_2NH_3	0.02	singlet	0
	BSr3-NH_3NH_2	0.63	singlet	0
BSr_3-N_2H_6	**1** BSr_3-NH_3NH_3	0.00	dublet	0.7695
	**2** BSr_3-NH_3NH_3	0.86	dublet	0.7524

2. Cartesian coordinates

Cartesian coordinates for the relaxed structures are provided below. Relative energies (E_r, in eV) are obtained for BSr_3–NH_yNH_x (x, y = 0–3) species with respect to the corresponding global minima (presented in Figure 3 of the main text).

BSr_3-NN, E_R=0 eV

			Coordinates
Atomic number	X	Y	Z
38	0.953578000	1.941414000	-0.242977000
38	0.962688000	1.937382000	-0.243183000
38	-2.413190000	0.004851000	-0.050239000
5	-0.350289000	0.002386000	1.578057000
7	0.905821000	-0.001557000	1.213693000
7	2.041973000	-0.004598000	0.571007000

BSr_3-NNH, E_R=0 eV

			Coordinates
Atomic number	X	Y	Z
38	-2.088047000	-0.843638000	-0.240403000
38	2.076126000	-0.871997000	-0.240357000
38	0.016617000	2.383564000	-0.007152000
5	0.000358000	0.133052000	1.500719000
7	-0.007874000	-1.127226000	1.177776000
7	-0.015038000	-2.159268000	0.285774000
1	-0.019856000	-3.041105000	0.792201000

BSr_3-NNH, E_R=1.72 eV

			Coordinates
Atomic number	X	Y	Z
38	-2.186526000	1.146219000	-0.300070000
38	0.003349000	-1.900898000	-0.241361000
38	2.646829000	0.832059000	-0.065421000
5	0.209048000	0.767183000	0.995795000
7	-0.778786000	-0.105167000	1.481169000
7	-1.771056000	-0.820753000	0.747301000
1	-0.815110000	-0.294908000	2.482100000

BSr_3-NNH_2, E_R=0 eV

			Coordinates
Atomic number	X	Y	Z
38	0.684736000	-2.158958000	-0.329829000
38	1.082158000	1.760239000	-0.537480000
38	-2.517656000	0.288349000	0.111493000
5	-0.129725000	-0.001431000	1.274703000
7	1.206090000	-0.286421000	1.187453000
7	2.224477000	0.689531000	1.453912000
1	3.124536000	0.226190000	1.516934000
1	2.039104000	1.153264000	2.340985000

BSr_3-NNH_2, E_R=0.93 eV

			Coordinates
Atomic number	X	Y	Z
38	0.997110000	-1.947341000	-0.481143000
38	1.002305000	1.944926000	-0.481369000
38	-2.466290000	0.002891000	-0.036701000
5	-0.091574000	-0.000116000	1.299473000
7	0.927956000	-0.000228000	2.506259000
7	1.350684000	-0.002312000	1.123158000
1	1.125233000	0.840190000	3.032601000
1	1.123435000	-0.839905000	3.034204000

BSr_3-NNH_3, E_R=0 eV

			Coordinates
Atomic number	X	Y	Z
38	-2.476147000	-1.040678000	0.044249000
38	1.914417000	-0.803694000	-0.694220000
38	-0.290324000	2.212929000	-0.064268000
5	-0.003874000	-0.165284000	1.343916000
7	-0.120913000	-1.196039000	0.566816000
7	3.298592000	-0.514711000	1.540745000
1	4.048179000	0.168756000	1.533657000
1	3.600697000	-1.293020000	2.118379000
1	2.504797000	-0.079224000	2.016547000

BSr_3-NNH_3, E_R=2.85 eV

			Coordinates
Atomic number	X	Y	Z
38	2.320867000	0.445570000	-0.418865000
38	-1.960464000	1.071145000	-0.480575000
38	-0.452439000	-2.195530000	0.115229000
5	0.026718000	0.171874000	1.256573000
7	-0.006673000	1.572783000	1.925300000
7	0.357276000	1.491817000	0.531067000
1	-0.881009000	2.061281000	2.137059000
1	1.059784000	-0.453286000	1.636141000
1	0.730788000	1.875407000	2.549368000

BSr_3-NNH_3, E_R=3.95 eV

			Coordinates
Atomic number	X	Y	Z
38	-2.931829000	-0.699714000	0.000634000
38	2.932051000	-0.116104000	0.000774000
38	-0.375104000	2.043672000	-0.000635000
5	-0.155044000	-0.842522000	-0.000272000
7	0.987927000	-1.578740000	-0.001801000
7	0.823702000	-3.038052000	-0.001531000
1	1.273275000	-3.447048000	0.827350000
1	1.269181000	-3.446668000	-0.832715000
1	-0.203103000	-3.234607000	0.000664000

BSr_3-N, E_R=0 eV

			Coordinates
Atomic number	X	Y	Z
38	-2.335019	-0.783854	-0.148334
38	2.109261	-1.140155	-0.149156
38	0.252018	2.160898	-0.042259
5	-0.009345	-0.132149	1.505183
7	-0.135879	-1.191575	0.769223

BSr_3-NH, E_R=0eV

			Coordinates
Atomic number	X	Y	Z
38	-2.357804	0.000575	-0.377004
38	1.427172	-1.800222	-0.175459
38	1.247002	1.921254	-0.036752
5	-0.206315	0.036409	1.429615
7	-1.354721	-0.525140	1.805477
1	-1.507424	-1.127130	2.603750

BSr_3-NH_2, E_R=0 eV

			Coordinates
Atomic number	X	Y	Z
38	-1.097405	-1.910925	-0.119091
38	2.195301	0.257613	-0.433546
38	-1.497368	1.720470	-0.059362
5	0.302669	-0.000158	1.506640
7	1.533699	-0.053344	1.982564
1	1.874767	-0.502434	2.821564
1	1.874767	-1.675356	-0.976737

BSr_3-NH_2, E_R=0.83 eV

			Coordinates
Atomic number	X	Y	Z
38	1.199660	1.862354	-0.250486
38	-2.254811	0.016063	-0.403418
38	1.168430	-1.880501	-0.248529
5	-0.110622	0.002712	1.482855
7	-0.393818	0.006887	2.864423
1	-0.496710	0.846066	3.410951
1	-0.498033	-0.828621	3.416263

BSr_3-NH_2, E_R=1.43 eV

			Coordinates
Atomic number	X	Y	Z
38	1.028736000	-1.807523000	0.044043000
38	1.025956000	1.808306000	0.044165000
38	-2.647085000	-0.001111000	-0.135194000
5	-0.534284000	-0.000829000	1.334097000
7	2.664265000	0.001786000	-0.501227000
1	3.504352000	0.001570000	0.077685000
1	3.028127000	0.002548000	-1.454073000

BSr_3-NH_2, E_R=1.55 eV

			Coordinates
Atomic number	X	Y	Z
38	2.372713	0.046565	-0.357229
38	-1.265912	1.762849	-0.274589
38	-1.166409	-1.816613	-0.263996
5	-0.114325	0.001895	1.490817
7	0.281647	0.027256	2.833025
1	1.239990	0.036472	3.156728
1	-0.374790	0.036841	3.598935

BSr_3-NH_3, E_R=0 eV

			Coordinates
Atomic number	X	Y	Z
38	1.038941	-1.881695	-0.160681
38	1.166176	1.669819	-0.315245
38	-2.516152	0.108164	-0.070219
5	-0.365496	0.029260	1.684584
7	0.887657	-0.027523	1.360289
1	2.473304	1.764114	2.139421
1	2.098581	-0.174553	-1.337024
1	2.861319	2.397843	2.006207

BSr_3-NH_3, E_R=0.69 eV

			Coordinates
Atomic number	X	Y	Z
38	1.339062	1.813729	-0.111425
38	-2.300028	-0.000265	-0.465565
38	1.339793	-1.813323	-0.111537
5	-0.248219	-0.000049	1.122923
7	-1.531153	-0.000487	1.960400
1	-1.653735	-0.813220	2.551341
1	-1.653854	0.812074	2.551515
1	0.871298	-0.000552	1.723736

BSr_3-NH_3, E_R=1.16 eV

			Coordinates
Atomic number	X	Y	Z
38	2.977521	0.461784	0.036282
38	-2.844697	0.842502	-0.009601
38	-0.302993	-1.856801	-0.066883
5	0.049819	1.033520	-0.052555
7	1.051435	1.900750	-0.217588
1	-1.299097	-0.414113	2.038823
1	-0.776754	0.046784	1.660609
1	0.933157	2.890048	-0.385865

BSr_3-NH_3, E_R=1.45 eV

			Coordinates
Atomic number	X	Y	Z
38	1.140062000	-1.794830000	-0.307367000
38	1.140102000	1.795214000	-0.305842000
38	-2.469681000	0.000241000	-0.163491000
5	-0.262206000	-0.000399000	1.373190000
7	1.080958000	-0.000182000	1.566049000
1	-0.161757000	-0.011081000	4.956780000
1	1.535965000	-0.000860000	2.472645000
1	-0.428201000	-0.008513000	4.256897000

BSr_3-NH_3, E_R=1.70 eV

			Coordinates
Atomic number	X	Y	Z
38	0.322672	2.123981	-0.079340
38	1.883841	-0.812352	-0.510416
38	1.960620	-1.182839	-0.101211
5	-0.089347	-0.316996	1.389395
7	-1.374877	-0.525630	2.101566
1	-1.449718	-1.353854	2.677880
1	-1.763542	0.257656	2.612987
1	-1.895025	1.466563	-0.692062

BSr_3-NH_3, E_R=2.55 eV

			Coordinates
Atomic number	X	Y	Z
38	1.428670	1.984146	0.048018
38	-1.996021	-0.001607	-0.682167
38	1.431344	-1.982583	0.048632
5	0.042443	0.000005	0.985324
7	-3.302645	0.000044	1.619645
1	-3.782702	-0.817492	1.980600
1	-3.783655	0.817777	1.978847
1	-2.359073	0.001032	2.026045

BSr_3-NH_3, E_R=2.73 eV

			Coordinates
Atomic number	X	Y	Z
38	0.042404	-0.000150	0.985478
38	-1.996046	0.002574	-0.682097
38	1.431620	1.982175	0.049008
5	1.428474	-1.984190	0.047622
7	-3.302824	-0.001805	1.619383
1	-3.783687	-0.820069	1.977595
1	-2.359303	-0.003047	2.025917
1	-3.783092	0.815246	1.981154

BSr_3-NH_3, E_R= 3.29 eV

			Coordinates
Atomic number	X	Y	Z
38	2.163398	0.855457	-0.000000
38	-0.915570	-0.866972	-1.841132
38	-0.915570	-0.866972	1.841132
5	-0.654648	1.072260	-0.000000
7	-0.046742	3.245246	-0.000000
1	-1.448412	2.967608	-0.818666
1	-1.448412	2.967608	0.818666
1	-0.915570	2.691534	-0.000000

BSr_3-NHNH, E_R=0 eV

			Coordinates
Atomic number	X	Y	Z
38	1.542800	-1.621758	-0.316188
38	0.251931	2.073452	-0.322047
38	-2.469398	-0.638309	0.026186
5	-0.196709	-0.192956	1.155231
7	1.088865	0.307372	1.494229
7	2.096802	0.642273	0.508771
1	1.380036	0.494395	2.448051
1	2.941170	0.914235	1.012676

BSr_3-$NHNH_2$, E_R=0 eV

			Coordinates
Atomic number	X	Y	Z
38	-1.630751	-1.762095	-0.261413
38	2.085138	0.272934	-0.792956
38	-1.372558	1.882723	0.172608
5	0.198902	-0.152670	1.075484
7	1.394751	-0.350229	1.814827
7	2.457763	-1.211846	1.352166
1	1.670131	0.146076	2.650445
1	2.065474	-2.146891	1.253491
1	3.192813	-1.256647	2.056637

BSr_3-$NHNH_2$, E_R=1.10 eV

			Coordinates
Atomic number	X	Y	Z
38	0.899518	-1.812105	-0.469938
38	0.899155	1.812295	-0.469930
38	-2.508120	-0.000212	0.070357
5	-0.276814	-0.000010	1.166719
7	1.110980	0.000241	1.978429
7	2.167436	-0.000197	0.959219
1	1.164872	0.809069	2.594794
1	1.164818	-0.807810	2.595806
1	3.064451	-0.000696	1.453670

BSr_3-$NHNH_3$, E_R=0 eV

			Coordinates
Atomic number	X	Y	Z
38	-2.310504	-1.286367	0.018327
38	1.880060	-0.777917	-0.706380
38	-0.802969	2.165031	-0.100822
5	0.067683	-0.106144	1.367750
7	1.082269	-0.900632	1.701289
7	3.747187	0.407836	0.792013
1	4.618320	-0.005765	1.104207
1	3.095793	0.383057	1.575904
1	3.932530	1.380714	0.578990
1	1.078431	-1.606094	2.426295

BSr_3-$NHNH_3$, E_R=0.06 eV

	Coordinates		
Atomic number	X	Y	Z
38	-2.751464000	-0.530365000	0.319210000
38	2.988608000	-0.574246000	-0.018536000
38	-0.065252000	2.060580000	-0.138955000
5	0.145142000	-0.639200000	0.756389000
7	1.009238000	-1.654310000	0.901958000
7	-1.584088000	-1.858612000	-1.686487000
1	-0.698680000	-2.127428000	-1.261440000
1	-1.357826000	-1.208379000	-2.434310000
1	-1.986325000	-2.682127000	-2.119811000
1	0.809167000	-2.522440000	1.379996000

BSr_3-$NHNH_3$, E_R=0.09 eV

	Coordinates		
Atomic number	X	Y	Z
38	2.227507000	0.160115000	0.313036000
38	-1.641422000	1.719653000	-0.410103000
38	-1.378737000	-1.939467000	0.026342000
5	-0.298488000	0.112775000	1.579653000
7	0.714007000	0.724247000	2.191305000
7	2.611299000	-0.443914000	-2.244981000
1	0.652646000	1.334802000	2.995695000
1	3.063721000	0.232828000	-2.852610000
1	1.625346000	-0.467524000	-2.509377000
1	2.994379000	-1.357760000	-2.468681000

BSr_3-$NHNH_3$, E_R=0.44 eV

	Coordinates		
Atomic number	X	Y	Z
38	-0.016150000	-0.000290000	1.431233000
38	-0.322829000	-0.002090000	3.043412000
38	1.056586000	-1.957737000	-0.453853000
5	1.048449000	1.962195000	-0.453085000
7	-2.313925000	-0.004421000	-0.369459000
7	1.352817000	0.002665000	1.141420000
1	-0.879894000	0.816278000	3.271575000
1	0.514455000	-0.004263000	3.631495000
1	-0.882687000	-0.819126000	3.269432000
1	2.056777000	0.003125000	1.880587000

BSr_3-$NHNH_3$, E_R=2.15 eV

	Coordinates		
Atomic number	X	Y	Z
38	-0.959788000	1.749098000	-0.495565000
38	-0.902072000	-1.766765000	-0.496399000
38	2.515464000	0.024935000	0.017647000
5	0.318932000	0.022995000	1.291251000
7	-1.094400000	-0.015404000	2.069575000
7	-2.090261000	-0.032597000	1.018228000
1	1.223585000	0.038363000	2.135478000
1	-1.197641000	0.788883000	2.685582000
1	-1.157026000	-0.832477000	2.674141000
1	-3.007935000	-0.049901000	1.457961000

BSr_3-$NHNH_3$, E_R=0 eV

	Coordinates		
Atomic number	X	Y	Z
38	1.062577	-1.657778	-0.400400
38	0.682354	2.003731	0.046765
38	-2.450738	-0.116525	-0.223453
5	-0.388468	-0.325232	1.429030
7	0.571646	-1.148892	2.208768
7	2.461553	0.366395	-0.649882
1	1.246365	-0.674529	2.794835
1	0.178577	-1.919327	2.734581
1	2.811533	0.538281	-1.592115
1	3.294111	0.440976	-0.065305

BSr_3-NH_2NH_3, E_R=0 eV

	Coordinates		
Atomic number	X	Y	Z
38	-2.142069	-1.318933	-0.048419
38	1.757727	-0.682207	-0.932197
38	-0.665672	2.163350	-0.153234
5	0.037454	-0.148562	1.321082
7	0.556586	-0.429923	2.626290
7	3.427831	-0.162457	1.078378
1	2.695749	-0.093673	1.787753
1	3.829860	0.764373	0.981919
1	4.154697	-0.772370	1.436659
1	0.402161	0.196665	3.401404
1	0.739878	-1.369488	2.940473

BSr_3-NH_2NH_3, E_R=0.02 eV

			Coordinates
Atomic number	X	Y	Z
38	1.549843000	-1.123058000	-0.517878000
38	-0.153889000	2.254149000	-0.296603000
38	-2.326194000	-1.028450000	-0.215567000
5	-0.256078000	-0.006426000	1.432584000
7	-0.338712000	-0.116315000	2.838447000
7	3.895124000	-0.340884000	0.482428000
1	4.782364000	-0.387944000	-0.011298000
1	0.190877000	-0.774976000	3.385724000
1	-0.939142000	0.459001000	3.406136000
1	4.074324000	-0.609739000	1.446231000
1	3.626186000	0.645848000	0.505993000

BSr_3-NH_2NH_3, E_R=0.63 eV

			Coordinates
Atomic number	X	Y	Z
38	1.087374000	-1.867342000	0.003343000
38	1.083687000	1.869066000	0.003115000
38	-2.306626000	-0.001917000	-0.597741000
5	-0.389733000	-0.000582000	1.351793000
7	-0.796638000	-0.000236000	2.955250000
7	1.657600000	0.001345000	-1.521783000
1	0.042705000	-0.003156000	3.519504000
1	-1.341165000	-0.820933000	3.198706000
1	-1.335123000	0.824431000	3.198489000
1	2.616598000	0.001372000	-1.869542000
1	1.090392000	0.000757000	-2.371640000

BSr_3-NH_3NH_3, E_R=0eV

			Coordinates
Atomic number	X	Y	Z
38	2.039026	-0.913190	-0.592840
38	-0.008573	2.465937	0.052182
38	-2.029130	-0.930300	-0.594981
5	0.000444	-0.019187	0.795400
7	-3.018555	-1.190901	1.845447
7	3.014256	-1.197329	1.850870
1	3.768057	-0.615967	2.201764
1	-2.139281	-0.726364	2.108890
1	-3.776626	-0.607276	2.183204
1	3.054478	-2.081901	2.345714
1	2.131585	-0.738393	2.112872
1	-3.060594	-2.069552	2.350608

BSr_3-NH_3NH_3, E_R=0.86eV

			Coordinates
Atomic number	X	Y	Z
38	1.966134000	-0.044219000	0.665401000
38	-0.743881000	2.138522000	-0.631508000
38	-1.519005000	-1.764459000	-0.502285000
5	-0.700180000	0.245600000	1.273471000
7	-1.532414000	-0.085683000	2.650012000
7	3.048824000	-1.228840000	-1.495845000
1	-0.924701000	-0.046406000	3.461718000
1	-1.970090000	-1.017405000	2.639413000
1	-2.276222000	0.591296000	2.787974000
1	2.565384000	-0.689175000	-2.213446000
1	2.728597000	-2.188030000	-1.602284000
1	4.039645000	-1.210718000	-1.721016000
